# The Week's Work

**Published:** 1910-10-29

**Authors:** 


### The Week's Work.

### THE LATE PRINCE FRANCIS OF TECK.

Not Middlesex Hospital only, for which he worked so well, but the entire hospital world of London loses a staunch friend and a keenly interested practical worker by the death of Prince Francis of Teck. His Serene Highness took a broad view of his responsibilities as a hospital official. He was never a mere figurehead, never again a mere advertisement. His services were sincere and really helpful, and not the least remarkable of his efforts was his attempt to get into touch with the hospital population of London. We need not draw attention to his work in the eastern districts of the Metropolis, but we are quite sure that that work in connection with various boys' clubs and institutions for young men made him realise to the full his responsibilities in the matter of hospitals. Too often hospital workers neglect this side of the business: they make no endeavours to reach their patients in the latter's everyday life, and they have consequently little or no knowledge of the conditions which prevail in the districts whence their institutions draw patients. Prince Francis's work in this connection is an example which should be followed more frequently, and it promised to make him one of the best-informed and most authoritative hospital chairmen in London.

### HOSPITAL ABUSE AND THE MINORITY REPORT.

The two papers, that by Mr. Finnie on Hospital Abuse and that by Mrs. Webb on a Unified Medical Service (which we publish in this number), togther with the discussions on them, provide interesting reading. We have thought it desirable to give these discussions more or less in full. There have appeared several abstracts of these papers and of the comments made upon them at the Glasgow Conference last month, but these share with abstracts in general the disadvantage that they do not give a complete picture of the arguments advanced for or against. The discussions on the papers have been most instructive, and let us add that they have also been most encouraging, for they have revealed this much at least, that there does not appear to be any subject of paramount importance in the hospital world which cannot be settled by mutual discussion. The two subjects dealt with in these papers may be classed among highly controversial problems of the day, and it was only natural that the opinions of speakers should show widely divergent methods of attacking these problems. Yet it is gratifying to notice that there is a general thread of agreement running through all the speeches. That speaks much for the solidarity of hospital workers, and for the future of the Association's work. No one can expect, or hope for, perfect unanimity on such matters as these outstanding problems of the day, for such agreement would stultify discussion, but it is perfectly legitimate to expect impartial survey and review of the whole question, and that is furnished by these discussions. We would specially draw attention to the comments by men with such wide opinions as Drs. Mackintosh and Raw, Mr. Anderson, and Professor Glaister, since they have dealt with the matter from various aspects.

### THE MINORITY REPORT AND THE CONFERENCE.

Perhaps it is a pity that the Conference passed no definite resolutions. On the other hand, it was scarcely to be expected that at its first meeting the Association should have committed itself to a specific line of opinion on matters which, as these discussions show, have given rise to much debate among members. Judging from a general review of all the speeches and comments it seems clear that the meeting was not enthusiastic about the Minority Report proposals. These proposals were very severely criticised, and more than one speaker pointed out that Mrs. Webb's paper showed that she was not acquainted with the methods and work in Scotland, but confined her strictures entirely to English Poor Law Administration. The Minority Report proposals found strong support from several members, mostly hailing from English hospitals, and the discussion on Dr. Loch's paper, which was in sum a representation of the case for the Majority Report, was animated and exhaustive. The prevailing opinion seemed to be that something more was wanted: that another set of proposals would meet with more united approval, and that both Majority and Minority suggestions left much to be desired. The references to the need for united action, the appeal to the German example by Mr. Thies, for instance, and the allusions to the schemes for the prevention of abuse in the discussion on Mr. Finnie's paper showed this very clearly. In the circumstances it is encouraging to find that there is such an amount of interest displayed, and that there is every desire on the part of hospital workers to deal with the question in a broad-minded, liberal spirit. That is the attitude which is imperatively necessary if we are to arrive at a definite conclusion which will help us to formulate a practical scheme. And it is not too much to say that the discussions at the Glasgow Conference have materially assisted in furthering the development of such a spirit at the present time.

### A BAD SYSTEM.

A most deplorable accident, which has resulted in the death of a four-year-old child from strychnine poisoning in the West Heath Fever Hospital (near Birmingham), has served at least to elicit a chorus of disapproval of the system, or lack of it, to which the tragedy is traceable. If such a scandalous state of affairs prevails in any other hospital in the kingdom it may be hoped that the lesson of what occurred at West Heath will suffice for its immediate abolition. Briefly, a visiting medical officer—whether senior or junior does not appear from the published accounts of the inquest—is entrusted with the dispensing, a method which prevails in many small hospitals where the exclusive services of a qualified dispenser would be but partially occupied. The doctor in question, desiring to repeat a mixture containing quinine and strychnine, placed a drachm of liquor strychninæ in the patient's empty bottle, and left it on a windowsill for the day sister of the ward to fill up with quinine solution from a stock bottle, of which she had custody. The sister failed to do this, and one of the nurses moved the bottle into the ward kitchen, which is apparently the accustomed place for medicines in this hospital; parenthetically, that system, too, may receive a word of condemnation. The night nurse found instructions in her report book to administer strychnine mixture, and accordingly gave the prescribed dose out of the nearly empty bottle, not realising that the previous stock had been entirely exhausted and that the bottle had gone to the dispensary to be refilled. The child thus received a large dose of strychnine, and died with symptoms of poisoning within twenty minutes of receiving it. The medical officer through whose default this dreadful mistake happened was censured by the jury, and it is impossible not to agree with the course thus taken. Seldom, surely, is such a disgracefully negligent manner of dispensing practised by any medical man; the whole incident is indefensible to the last degree, and reflects discredit on those responsible for it. Doubtless, such a catastrophe will be effectually guarded against in the future at West Heath; but in any ordered hospital it should be quite impossible for it to happen.

### THE NEWCASTLE INFIRMARY CHAPEL.

The latest stage of this dispute was reached on Saturday last, when a Court of Governors was held in the out-patient department to consider the refusal of the House Committee to carry out the Governors' instructions to open the chapel to Church of England and Nonconformist services on equal terms. There came before the meeting, which does not seem to have been free from the well-known horrors of religious controversy, three proposals. Mr. Newbigin advocated that the holding of religious services by an accredited Free Church minister should be authorised, adding that he did not believe an injunction in restraint would be granted if applied for. This proposal does not appear to have been voted upon, although what is described as a " storm of dissent " was raised by the speech of a governor to the effect that many Churchpeople would withdraw their subscriptions if the original consecration of the chapel were interfered with. A motion to keep the chapel closed was defeated. The Lord Mayor of Newcastle then proposed that a committee should be appointed, representing the governors, to instruct two firms of solicitors, the one for the governing body, the other for the Nonconformists. These solicitors should then draw up a plain statement of fact, and make a joint appeal to the High Court of Justice to see if the original consecration of the chapel, according to the rites of the Church of England solely, had been legal or illegal. He stated that the cost, which he volunteered to bear personally, would be between £200 and £300. In the end this proposal was adopted by a large majority, and a committee appointed with the Lord Mayor as chairman.

### THE NECESSITY FOR TOLERATION.

It is to be hoped that progress towards settlement of the trouble has been made, but to the onlooker it would appear that no measures can be really successful which are not largely concerned with the equity of the question. Undoubtedly whoever first insisted on a certain ecclesiastical consecration entailing exclusion of Nonconformists from worshipping after their own tenets in the chapel were very ill-advised persons, shadowy legal rights notwithstanding. Modern England, let alone a modern democratic industrial community, is not to be ruled according to the extreme ideas, say, of High Churchmen. It must be remembered, too, that in the class from which hospital patients are drawn there are many more Nonconformists than adherents of the Church of England, and, as appears abundantly from what has fallen from speakers in discussion on the present topic, a good few Agnostics and Freethinkers. Hospitals have no money to spare to provide separate accommodation for the sects. We are glad to see that a gentleman who was going to move on this occasion to spend £5,000 in building a Nonconformist chapel had the unselfishness and good sense to withdraw his proposal. Otherwise patients who might have been cared for with this money would have had good cause to exclaim, like wounded Mercutio, " A plague o' both your houses! " Again, though we cannot pretend to a close knowledge of the exigencies of the internal politics of the Newcastle Infirmary management, yet it seems sound sense to affirm that the Governors should be masters in their own house, and that, having once issued instructions to a subaltern body, it would be better for their prestige if such instructions were carried out, distinguished and valuable workers though the objectors to these instructions may have been. Hospital authorities who have watched this dispute will be more than ever impressed with the necessity of practical views, toleration, and moderation in providing for the religious requirements of their patients.

### THIS WEEK'S DRUG MARKET.

A very fair amount of business has been done during the week as compared with the previous week, but there have not been many noteworthy alterations in prices. At the recent public sale of drugs large quantities of senna were disposed of at previous rates, and rather higher prices were paid for Cape aloes. Grey Jamaica sarsaparilla is about 2d. per lb. dearer. Rhubarb, notwithstanding the low prices which have been ruling for some time, is still tending somewhat cheaper. A further advance in the price of cocaine is considered not unlikely, although there have been two advances quite recently. Oil of cloves is dearer. It is thought probable that higher prices will shortly be asked for saffron. Chiretta, sugar of milk, and ergot of rye are all tending dearer. Higher prices are asked for potassium permanganate. Buchu leaves are again rather lower in price. Cream of tartar has again been slightly advanced in price.

